# Influenza H5Nx viruses are susceptible to MEK1/2 inhibition by zapnometinib

**DOI:** 10.1080/22221751.2025.2471022

**Published:** 2025-02-20

**Authors:** André Schreiber, Nicole Oberberg, Benjamin Ambrosy, Franziska Rodner, Sriram Kumar, Duygu Merve Caliskan, Linda Brunotte, Martin Beer, Stephan Ludwig

**Affiliations:** a Institute of Virology Muenster, University of Muenster, Muenster, Germany; b Institute of Diagnostic Virology, Friedrich-Loeffler-Institut, Greifswald-Insel Riems, Germany

**Keywords:** HPAIV, antiviral drug treatment, Raf/MEK/ERK signalling pathway, MEK1/2 inhibition, Zapnometinib

## Abstract

Highly pathogenic avian influenza A viruses (HPAIV) pose a significant threat to both animal and human health. These viruses have the potential to cause severe respiratory and systemic infections in birds and several mammalian species. The recent global outbreak of the H5N1 clade 2.3.4.4b spread in wild and domestic birds is now considered to be a panzoonosis. Spillover events in dairy cattle farms in the U.S. have highlighted the urgent need for effective antiviral therapies, especially in view of human infections. This study investigates the selective MEK1/2 inhibitor zapnometinib (ZMN) as a potential antiviral agent against HPAIVs. Our *in vitro* experiments demonstrate that ZMN significantly impairs viral replication across multiple HPAIV strains, including H5N1 clade 2.3.4.4b in cell lines and primary bronchial epithelial cells. The mechanism of action is based on the nuclear retention of newly produced viral ribonucleoprotein complexes (vRNP), when the MEK/ERK/RSK1 kinase cascade is inhibited. We furthermore could show, that ZMN not only acts antiviral in a standalone treatment but has synergistic potential when used in combination with direct-acting antivirals like oseltamivir or baloxavir. Therefore, ZMN treatment offers a promising strategy for future antiviral development.

## Introduction

Avian influenza viruses (AIV), in particular H5 and H7 subtypes, represent a zoonotic risk for public health. The primary natural reservoir of H5 and H7 AIV is wild waterfowl but they can also infect gallinaceous poultry. On top of that these viruses can cross species barriers, infecting mammals, like minks, pigs, humans and more recently also cows [1]. Especially infection of poultry results in the evolvement of highly pathogenic AIV (HPAIV), upon acquisition of a multibasic cleavage site (MBCS) within the haemagglutinin (HA) enabling the cleavage by the host protease furin [2]. HPAIV subtype H5N1 (A/Goose/Guangdong/1/1996 (GsGD)) was first detected in domestic waterfowl in 1996 in Southern China (WHO). In 1997, H5N1 was discovered for the first time in humans during a poultry outbreak in Hong Kong [3]. According to the WHO, from 2003 to the end of 2024 a total number of 954 cases and 464 deaths have been reported for H5N1 in humans worldwide, indicating that so far the transmissibility from animals to humans is low, but the case fatality rate is high [4]. Since 2021 the HPAI Eurasian strain H5N1 clade 2.3.4.4b started to spread severely in domestic and wild birds all around the world, now considered to be a panzoonosis [5,6]. Increasing numbers of zoonotic spillover events were reported for minks in Spain [7]*,* marine mammals including dolphins and sea lions in South America [8] and within dairy cattle farms in the U.S. [9], posing the risk that the virus evolves into a strain easily transmissible between mammals. In addition, evidence of mammalian adaptation could be found in infected seals [10].

Besides vaccination-induced immunization as a prophylaxis intervention, antiviral drugs administered to treat viral infections are the second strategy to lower the risks of mortality and progression to critical as well as fatal conditions. All licensed drugs, such as oseltamivir or baloxavir interfere directly with viral components (direct-acting antivirals – DAAs). More recently, drugs that interfere with cellular pathways crucial for viral replications (host targeted antivirals – HTA) were developed and have now partially entered clinical trials in patients, like the OAT3 inhibitor Probenecid [11], the sialidase DAS-181 [12,13] (NCT01037205) or the calcium-channel blocker Diltiazem [14] (NCT03212716). Prolonged treatment with DAAs is accompanied by resistance selection, a risk that can be minimized by using HTAs [15]. The HTA zapnometinib (ZMN) a small molecule inhibitor of the two isoforms MEK1 and MEK2 was recently reported to have antiviral properties against SARS-CoV-2 [16] and human influenza strains [17]. As a second beneficial effect, it reduces the expression of proinflammatory cytokines and chemokines thus avoiding a severe hyperinflammatory cytokine storm, without interfering with the antiviral interferon response [16]. In addition, it was shown that ZMN acts synergistically with DAAs and has a low risk of resistance selection [15,18], which makes this inhibitor a particularly interesting and promising drug against SARS-CoV-2 and human influenza virus infections. Especially with regard to the increasingly emerging H5N1 strains with reduced susceptibility to DAAs like oseltamivir [19,20]. Meanwhile, ZMN has entered the clinical stage of development. Two phase 1 clinical trials in 2019 (NCT04385420) and 2023 (NCT05555823) have demonstrated that the drug is safe and very well tolerated in humans. Moreover, in the phase 2b clinical proof-of-concept trial “RESPIRE” (NCT04776044), a clinically relevant efficacy of ZMN was demonstrated in hospitalized patients with COVID-19 (WHO clinic severity scale stage 4) [21]. Thus, the drug has passed a major hurdle in clinical development and may enter clinical practice in the near future.

In this study, we elucidate the antiviral efficacy of ZMN against HPAIV including H5N1 clade 2.3.4.4b viruses, either alone or in combination with DAAs.

### Material and methods

#### Cells

Human alveolar type II epithelial cells (A549), human lung adenocarcinoma cells (Calu-3) and African green monkey kidney cells (VeroE6) were cultured in Dulbecco´s Modified Eagle´s Medium (DMEM) (Sigma-Aldrich). Madin-Darby canine kidney cells (MDCK II) were cultured in Minimum Essential Medium (MEM) (Sigma-Life Science). Both media were supplemented with 10% (v/v) fetal bovine serum (FBS) (Capricorn Scientific). Colon carcinoma cells (CaCo2) were cultured in Dulbecco´s Modified Eagle´s Medium (DMEM) supplemented with 10% (v/v) FBS and 1% (v/v) Non-essential Amino acids (Capricorn Scientific). Primary human bronchial epithelial cells (HBEpC) (PromoCell) were cultured in airway epithelial growth medium (PromoCell) supplemented with 4 µL/ml bovine pituitary extract, 10 ng/ml epidermal growth factor, 5 µg/ml insulin, 0.5 µg/ml hydrocortisone, 0.5 µg/ml epinephrine, 6.7 ng/ml triiodo-L-thyronine, 10 µg/ml transferrin and 0.1 ng/ml retinoic acid. Experiments with primary cells were conducted at passage number 3. Cells were cultivated at 37 °C and 5% CO_2_ under humidified conditions.

#### Viruses

All HPAIV experiments were performed in a laboratory approved for work under biosafety level (BSL) 3. The avian influenza viruses A/Thailand/KAN-1/2004 (H5N1) (Thailand (H5N1)) (clade 1), A/Vietnam/1203/2004 (H5N1) (Vietnam (H5N1)) (clade 1), A/Mallard/Bavaria/1/2006 (H5N1) (Mallard (H5N1)) (clade 2.2.1), A/FPV/Bratislava/79 (H7N7) (FPV (H7N7)), A/Anhui/1/2013 (H7N9) (Anhui (H7N9)), the recombinant mouse-adapted laboratory strain SC35M (H7N7) and the human strain A/Puerto Rico/8/1938 (H1N1) (PR8 (H1N1)) were taken from the strain repository of the IVM, Münster. The clade 2.3.4.4b viruses A/chicken/Germany-NI/AI01599/2021 (H5N1) (Chicken (H5N1)), A/red knot/Germany-SH/AI03424/2020 (H5N3) (Red knot (H5N3)), A/gull/Germany-SH/AI01498/2021 (H5N4) (Gull (H5N4)), A/turkey/Germany-SH/R425/2017 (H5N5) (Turkey (H5N5)), A/grey heron/Germany-SN/R572/2017 (H5N5) (Grey heron (H5N5)), A/buzzard/Germany-MV/AI02166/2020 (H5N5) (Buzzard (H5N5)), A/domestic duck/Germany-MV/AR613-L02727/2018 (H5N6) (Domestic duck (H5N6)), A/tufted duck/Germany/AR8444-L01986/2016 (H5N8) (Tufted duck (H5N8)), A/greylag goose/Germany-NI/AR703-L02138/2017 (H5N8) (Greylag goose (H5N8)), A/black swan/Germany-BW/R1364/2017 (H5N8) (Black swan (H5N8)), A/white fronted goose/Germany-BB/AI00018/2020 (H5N8) (White fronted goose (H5N8)) and A/barnacle goose/Germany-SH/AI02167/2020 (H5N8) (Barnacle goose (H5N8)) were kindly provided by the Friedrich Loeffler Institute (FLI), Greifswald-Riems. All viruses were propagated on MDCK II cells in infection MEM (MEM supplemented with 1% Penicillin/Streptomycin (P/S), 0.2% (v/v) bovine serum albumin (BSA) (35%), 0.01% MgCl_2_, 0.01% CaCl_2_).

#### Virus infection

Virus dilutions were prepared in infection PBS (PBS supplemented with 1% P/S, 0.2% (v/v) bovine serum albumin (BSA) (35%), 0.01% MgCl_2_, 0.01% CaCl_2_). Cells were infected for 0.5 h at 37 °C, followed by a washing step with PBS and incubation in infection DMEM (DMEM supplemented with 1% P/S, 0.2% (v/v) BSA, 0.01% MgCl_2_, 0.01% CaCl_2_). Primary HBEpCs were infected with airway epithelial growth medium for 0.5 h at 37 °C, followed by a washing step with PBS and incubation in airway epithelial growth medium.

#### Inhibitors

Zapnometinib (ZMN) was obtained from Atriva Therapeutics GmbH. Oseltamivir carboxylate (OTC) was purchased from Santa Cruz Biotechnology and baloxavir acid (BXA) from MedChem Express. ZMN and BXA were dissolved in DMSO (Roth, Sigma), and OTC was dissolved in ddH_2_O.

#### Virus titration by plaque assay

MDCK II cells were grown to confluent monolayers in 12-well plates and infected with 10-fold virus dilutions in infection PBS for 0.5 h at 37 °C, followed by replacement with plaque medium (14.2% (v/v) 10 MEM, 0.3% (v/v) NaHCO_3_ (7.5%), 0.014% (v/v) DEAE-dextran (1%), 1.4% (v/v) 100x P/S, 0.3% (v/v) BSA (35%), 0.01% (v/v) CaCl_2_ (1%), 0.01% (v/v) MgCl_2_ (1%)), 0.9% (v/v) Agar (3%), 0.15 µg TPCK-treated trypsin (Sigma). Cells were incubated at 37 °C until plaques were countable.

#### Indirect immunofluorescence

A549 cells seeded in 15 µ-slide chambers (ibid) were washed twice with PBS followed by 10-min 4% PFA fixation and permeabilization at 4 °C using 70% EtOH overnight. Samples were washed with PBS and blocked with 3% BSA in PBS for 1 h at room temperature, followed by a 1 h incubation with anti-NP antibody (BioRad) diluted in blocking buffer for 1 h at room temperature. After three times washing with blocking buffer, samples were incubated with a secondary antibody diluted in blocking buffer for 45 min at room temperature. Nuclei were stained with DAPI. Samples were washed twice with PBS followed by 10 min 4% PFA fixation.

#### Fluorescence *in situ* hybridization

Fluorescence *in situ* hybridization was conducted as described before [22]. Indirect immunofluorescence stained samples were rehydrated with wash buffer (10% formamide, 2x SSC in DEPC treated water) for 10 min at room temperature. Samples were 90 min blocked in hybridization buffer (10% dextran sulfate, 2 mM Vanadyle-ribonucleoside complex, 0.02% RNA-free BSA, 1 mg/ml *E.Coli* tRNA, 2x SSC, 10% formamide in DEPC treated water) at 28 °C. 48 segment-specific PB2-vRNA probes (Quasar®570) were diluted in hybridization buffer (2.5 µM) and samples were incubated overnight at 28 °C. After three washing steps with wash buffer, samples were post-fixated with 4% PFA for 10 min and washed three times with PBS and twice with ddH_2_O. Epifluorescence analysis was conducted with the Axiovert 200M microscope and the AxioVision V4.8.2.0 software (Zeiss). 5 images were captured for each virus, condition and experiment. The images were taken randomly without selecting or searching for optimal areas to ensure unbiased representation. Shown are representative epifluorescence pictures of single focal planes.

#### Quantification and statistical analysis

The AxioVision V4.8.2.0 software (Zeiss) was used to format the immunofluorescence pictures. Depicted graphs and statistical analysis were prepared with GraphPad PRISM version 8.4.3 (GraphPad Software). Drug combinatory screening was evaluated and visualized using the open-source web application SynergyFinder Plus [23]. Sample size and statistical tests are included in the respective figure legends.

## Results

### Zapnometinib inhibits the replication of hpaiv h5n1 and clade 2.3.4.4b h5nx viruses

**Figure 1.**
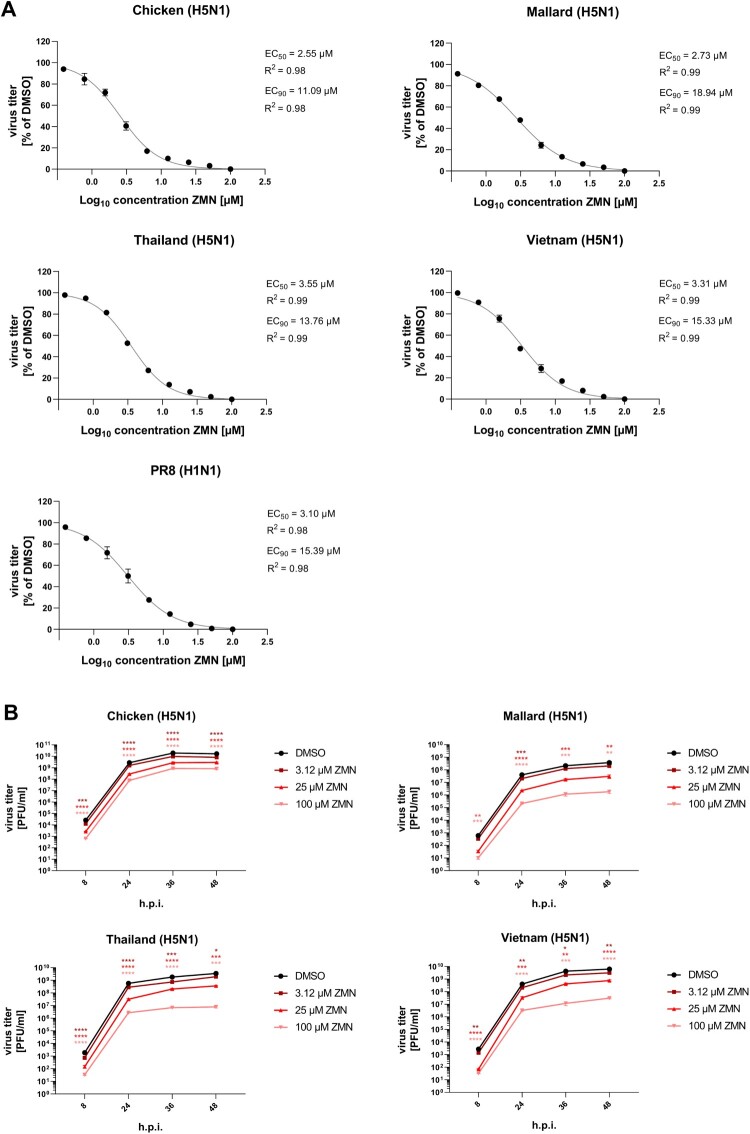
Antiviral activity of ZMN against different H5N1 viruses. Note: A549 cells were infected with different H5N1 viruses and PR8 (H1N1) (MOI: 0.01). At 0.5 h.p.i. ZMN treatment was initiated. (A) Viral titres were analysed 24 h.p.i. and EC values were determined. Data represent means ± SEM of three independent experiments, each performed in triplicates. (B) Viral titers were analysed after the indicated time points. DMSO treated cells served as negative control. Data represent means ± SEM of three independent experiments, each performed in triplicates. Significance was calculated using a one-way ANOVA in combination with a Dunnett´s multiple comparisons test with DMSO as reference for each time point individually (*p ≤  0.0332; **p ≤ 0.0021; ***p ≤ 0.0002; ****p ≤  0.0001). See also Figure S1.

We previously showed that MEK1/2 inhibition dampens the replication of human and avian IAV [17,24–26]. Based on these data the aim of this study was to evaluate if the specific MEK1/2 inhibitor zapnometinib (ZMN), which is under clinical development as an anti-infective drug, would also display an antiviral activity against recent highly pathogenic avian influenza viruses. For this purpose, the inhibitory capacity of ZMN against H5N1 strains of distinct H5 clades (A/Chicken/Germany/2021 (Chicken (H5N1)) clade 2.3.4.4b, A/Mallard/1/Bavaria/2006 (Mallard (H5N1)) clade 2.2.1, A/Thailand/KAN-1/2004 (Thailand (H5N1)) clade 1, A/Vietnam/1203/2004 (Vietnam (H5N1)) clade 1) was evaluated in the human alveolar type II epithelial cell line A549 in a non-cytotoxic range (Figure 1A, Figure S1A-C). We found a significant reduction of progeny viral titres 24 h.p.i. for all tested H5N1 viruses in a concentration range of 0.78–100 µM, with only slight variations. The 50% effective concentrations (EC_50_) were between 2.55 and 3.55 µM and the EC_90_ between 11.09 and 18.94 µM, which is comparable with the inhibitory potency against the human strain PR8 (H1N1), for which we calculated an EC_50_ of 3.10 µM and an EC_90_ of 15.39 µM (Figure 1A, Figure S1A-B). The selectivity indices (SI), a ratio of the 50% cytotoxicity and the biological activity (50% effective concentration) are between 121.58 and 169.25 for the H5N1 strains and 139.23 for PR8 (H1N1) (Table 1). These data demonstrate a subtype-independent inhibitory capacity of ZMN in non-toxic concentrations. Importantly, the SI of ZMN against IAV evaluated in this study are in the same range as those for other HTAs, like the DHODH inhibitors Brequinar, Teriflunomide, S312 or S416 [27,28], the receptor tyrosine kinase inhibitors Tyrphostin A9 or AG879 [29], the IMPDH inhibitor N30 [30], the Akt inhibitor MK2206 [31] or the XPO1 antagonist Verdinexor [32], showing that ZMN achieves an effective anti-influenza activity without disproportionately harming host cells, making it competitive with other HTAs.

**Table 1.** *In vitro* antiviral activity of ZMN against different H5N1 viruses.

Virus	Strain	EC_50_ (µM)	EC_90_ (µM)	SI_EC50_
H5N1	Chicken	2.55	11.09	169.25
	Mallard	2.73	18.94	158.10
	Thailand	3.55	13.76	121.58
	Vietnam	3.31	15.33	130.39
H1N1	PR8	3.10	15.39	139.23

Note: Shown are the EC values of ZMN (Figure 1A). The 24 h CC_50_ (Fig. S1C) was used to calculate the selectivity indices (SI).

To analyse whether these findings are not due to a simple delay of replication and would represent a robust inhibition over time we evaluated the inhibitory potency of ZMN in multi-replication cycle growth kinetics from 8 h.p.i. to 48 h.p.i., analysing the drug concentrations 3.12, 25 and 100 µM on the aforementioned H5N1 strains. All growth kinetics showed a significant reduction of progeny viral titres up to 48 h.p.i., with the exception of Mallard (H5N1) treated with 3.12 µM ZMN. These results are caused by the antiviral mode of action and not a cytotoxic side effect of ZMN (Figure 1B, Figure S1D).

**Figure 2.**
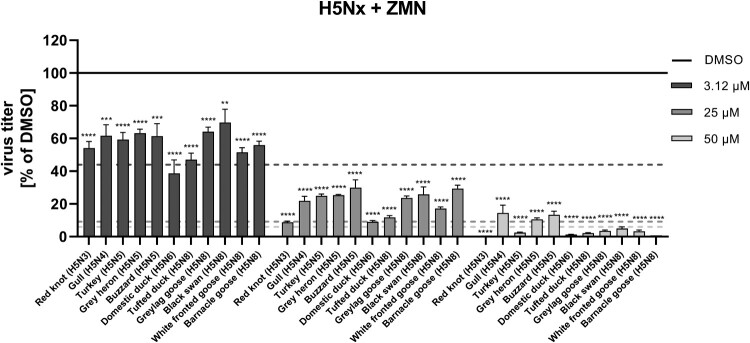
Susceptibility of H5Nx viruses to ZMN treatment. Note: A549 cells were infected with different H5Nx viruses (MOI: 0.01). At 0.5 h.p.i. ZMN treatment was initiated. Viral titers were analysed 24 h.p.i. and are depicted in % of DMSO. DMSO was arbitrarily set to 100% (black line). Data represent means ± SEM of three independent experiments, each performed in triplicates. Significance was calculated using a one-way ANOVA in combination with a Dunnett´s multiple comparisons test with DMSO as reference for each virus individually (**p ≤ 0.0021; ***p ≤ 0.0002; ****p ≤ 0.0001). Colour-coded dashed lines indicate relative titer reduction evaluated for Chicken (H5N1) for the respective ZMN concentrations. See also Figure S2.

Besides testing subtype H5N1 viruses, we furthermore wanted to evaluate, if ZMN has an even broader inhibitory potential against different H5Nx viruses. The clade 2.3.4.4b H5Nx strains used in this study were isolated from acute infections of livestock or wild birds. The strains were selected to represent the spectrum of the most prevalent subtypes and genotypes of circulating H5Nx viruses in Germany, including H5N3, H5N4, H5N5, H5N6 and H5N8. All tested viruses replicated to titres between 10^6^ and 10^9^ PFU/ml in A549 cells within 24 h, indicating that the effects on viral replication caused by ZMN treatment were tested under conditions of high replication (Figure S2). Highest titers in a drug-free environment (DMSO) were found for Buzzard (H5N5) (5.0·10^8^ PFU/ml), while we obtained titers in the 10^7^ log range for Gull (H5N4) (5.4·10^7^ PFU/ml), Domestic duck (H5N6) (5.6·10^7^ PFU/ml), Tufted duck (H5N8) (3.5·10^7^ PFU/ml) and White-fronted goose (H5N8) (4.7·10^7^ PFU/ml) and titres in the 10^6^ log range for Red knot (H5N3) (4.7·10^6^ PFU/ml), Grey heron (H5N5) (5.2·10^6^ PFU/ml), Turkey (H5N5) (2.0·10^6^ PFU/ml), Greylag goose (H5N8) (4.7·10^6^ PFU/ml), Black swan (H5N8) (3.8·10^6^ PFU/ml) and Barnacle goose (H5N8) (8.4·10^6^ PFU/ml). Viral replication of all tested H5Nx viruses was significantly inhibited for all ZMN concentrations (3.12, 25, 50 µM) (Figure 2, Figure S2), however, slight differences in the inhibitory potency of ZMN on different isolates should be mentioned. Most effective titre reductions occurred for the Red knot (H5N3), Domestic duck (H5N6) and Tufted duck (H5N8) viruses, while Gull (H5N4), Grey heron (H5N5) and Buzzard (H5N5) were the least susceptible subvariants. Titre reductions for 3.12 and 50 µM were comparable to the H5N1 viruses, but most H5Nx viruses showed a lower susceptibility to 25 µM ZMN. Collectively, these experiments indicate the dependence of H5 viruses on the activation of the Raf/MEK/ERK/RSK1 signalling cascade.

### ZMN causes nuclear retention of newly produced vRNP complexes

**Figure 3.**
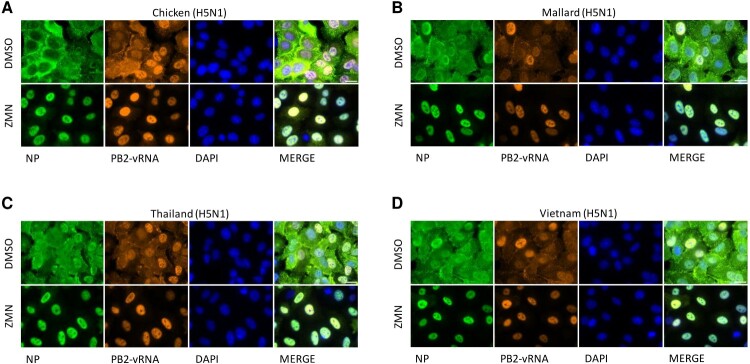
Nuclear retention of vRNP complexes after ZMN treatment. Note: A549 cells were infected with different H5N1 viruses (MOI: 5). At 3 h.p.i. ZMN treatment (50 µM) was initiated. At 9 h.p.i. Samples were prepared for immunofluorescence analysis of NP protein and PB2-vRNA. Shown are representative images of three independent experiments. (Scale bar: 20 µm). See also Figure S3.

We could previously show, that influenza viruses activate the Raf/MEK/ERK/RSK1 signalling cascade in the late stage of the infection cycle to promote the phosphorylation of S269 and S392 of the nucleoprotein (NP). This provides a binding site for the viral matrix protein 1 (M1), a crucial step in the formation of the vRNP export complexes [22]. Comparison of S269/S392 (S327/S448 in influenza B) revealed structural homologies between the respective influenza A/H5N1, A/H1N1 and influenza B residues (Figure S5). A protein blast of more than 65,000 sequences of human, avian, swine and bat influenza viruses using the NCBI Influenza Database Nucleotide BLASTp alignment tool identified no polymorphisms for the residue S392 and 10 polymorphisms for S269 (Table S3). This high conservation of the two residues indicates the importance for the viral life cycle. Phosphorylation of S269/S392 can be blocked by MEK1/2- or RSK-specific inhibitors [22]. We conducted single-cycle infection experiments with highly pathogenic H5N1 strains and treated the cells 3 h.p.i. with 50 µM ZMN, a concentration that showed significant titre reduction of approx. 95% or one log unit on the production of progeny viral particles, to analyse if the inhibitor treatment causes a nuclear retention of the viral genome. To ensure that the cellular localization of the vRNP complexes was analysed, we co-stained the viral NP, as the major protein component of the vRNPs, together with the vRNA genome itself. The immunofluorescence analysis revealed a cytoplasmic NP and vRNA signal in the DMSO control treated samples, after a total infection time of 9 h, indicating that the nuclear export of newly produced vRNPs did take place at that time point. In comparison, protein and vRNA signals could be detected exclusively in the nucleus after MEK1/2 inhibition, clearly demonstrating that ZMN blocks the nuclear export of H5N1 vRNPs (Figure 3). The qualitative analysis did not show differences in the nuclear retention between the H5N1 viruses, which is in line with the comparable EC values (Figure 1A, Table 1). This effect was also found for HPAIV of the subtype H7 (FPV (H7N7), SC35M (H7N7), Anhui (H7N9)) (Figure S3A). In addition, these viruses showed titre reductions after 24 h that are comparable to the H5 viruses, indicating that the inhibitory capacity of ZMN is broadly directed against influenza viruses, independently of the subtype (Figure S1A, B; S3B, C).

### ZMN acts antiviral in different cell lines and HBEpCs and has a synergistic potency with licensed direct-acting antivirals (DAAs)

**Figure 4.**
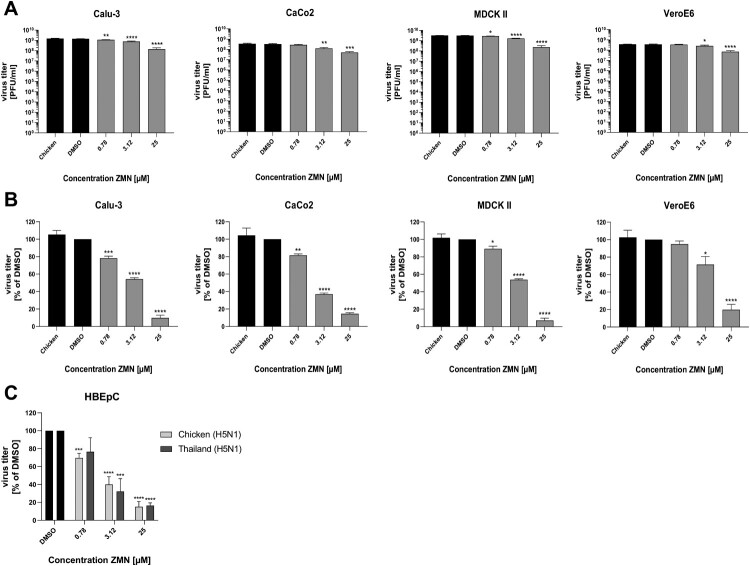
Antiviral properties of ZMN against Chicken (H5N1) in different cell lines. Note: Different cell lines (A, B) or HBEpCs (C) were infected with Chicken (H5N1) (A, B: MOI: 0.01; C: MOI: 0.1) or Thailand (H5N1) (C: MOI: 0.1). ZMN treatment was initiated 0.5 h.p.i. Viral titers were analysed via plaque titration 24 h.p.i. (A) Absolute viral titers in PFU/ml. (B, C) Relative viral titers in % of DMSO. DMSO was arbitrarily set to 100%. See also Table S2. (A–C) Shown are the results of three independent experiments, each performed in triplicates. Significance was calculated using a one-way ANOVA in combination with a Dunnett´s multiple comparisons test (*p ≤  0.0332; **p ≤  0.0021; ***p  ≤  0.0002; ****p  ≤ 0.0001). (C) Significance calculation was performed for each virus individually. See also Figure S4.

We could previously demonstrate, that ZMN has anti-SARS-CoV-2 properties in Calu-3 and CaCo2 cell lines [16]. Therefore, the question has been raised if the MEK1/2 inhibitor has a cell line-independent inhibitory effect on influenza virus replication. We used the human lung adenocarcinoma cell line Calu-3, the colon carcinoma cell line CaCo2, the canine kidney cell line MDCK II and the African green monkey kidney cell line VeroE6, all susceptible to influenza virus contagions and infected them with the H5N1 strains Chicken (H5N1) (Figure 4A, B) or Thailand (H5N1) (Figure S4A, B). Both viruses replicated to high titres in all tested cell lines, with the highest replication efficacies in MDCK II cells, followed by Calu-3, VeroE6, and CaCo2 cells. To evaluate the inhibitory capacity of ZMN, we tested three drug concentrations (0.78, 3.12, 25 µM) which showed approx. 10%, 50% and 90% titre reduction within the EC evaluation (Figure S1B). Antiviral efficacies of the tested drug concentrations were comparable to A549 cells (Table S2). To confirm our results in a more biologically relevant human cell model we used primary human bronchial epithelial cells (HBEpCs) which were infected with Chicken (H5N1) or Thailand (H5N1). Both viral strains replicated successfully in the primary cells reaching titres of 1.0·10^6^ and 1.7·10^7^ PFU/ml, respectively. We found a comparable inhibitory potential in HBEpCs using 0.78, 3.12 and 25 µM ZMN, which was in a range not cytotoxic for the cells (Figure 4C, Figure S4C, E). Titres were reduced by approx. 20%, 60% and 80% for both tested viruses. These experiments revealed a cell line-independent antiviral mode of action of ZMN against H5N1 viruses, which is not restricted to permanent cell lines but could also be found for primary cells.

**Figure 5.**
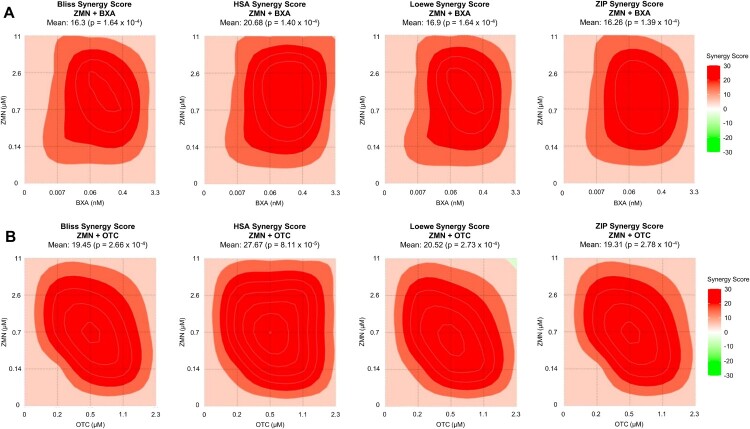
Evaluation of the synergistic potential of ZMN with the DAAs BXA or OTC. Note: A549 cells were infected with Chicken (H5N1) (MOI: 0.01). Drug treatment was initiated 0.5 h.p.i. Viral titers were determined by plaque titration 24 h.p.i. Bliss independence (Bliss), Loewe additivity (Loewe) highest single agent (HSA) or Zero Interaction Potency (ZIP) reference models were used to evaluate synergism. Shown is a colour-coded 2D contour visualization with red indicating synergistic interactions while green indicates antagonistic interactions. See also Figures S6–S8.

Synergistic drug combinations increase the capacity of antiviral therapies. In a former study, we could show that ZMN possesses such a synergistic mode of action against SARS-CoV-2 when combined with the direct-acting antiviral drugs (DAAs) remdesivir or nirmatrelvir [18]. Furthermore, it was shown, that other MEK1/2 inhibitors do act synergistic with oseltamivir against human IAVs [26]. To test if the combination of ZMN with direct anti-influenza drugs results in a potentiated inhibitory effect, we combined the NA inhibitor oseltamivir carboxylate (OTC) or the cap-dependent endonuclease inhibitor baloxavir acid (BXA) with ZMN. These two DAAs with different modes of action were tested to determine whether ZMN generally acts synergistically with DAAs against H5N1, or if only specific combinations are synergistic. Chicken (H5N1) was susceptible to both DAAs in a non-cytotoxic range comparable to the literature (Figure S6 A-H) [33,34]. To determine a synergistic mode of action of ZMN with BXA or OTC, A549 cells were infected with Chicken (H5N1) and either treated with the single drugs (drug + DMSO) or the combinations ZMN + BXA and ZMN + OTC in a non-cytotoxic range (Figure S6I, J). The viral titres of the controls (untreated and DMSO-treated) exceeded 5·10^9^ PFU/ml, indicating that the experiments were conducted under high-replicating conditions (Figures S7A and S8A). We used drug doses resulting in approx. 1%, 10%, 50% and 90% titre reductions, thereby screening concentration ranges beginning at amounts that are not effective as monotherapies. Yet, even the non-effective lowest single drug concentrations (0.007 nM BXA, 0.2 µM OTC, 0.14 µM ZMN) resulted in more than 20% titre reductions in the combinational treatment (ZMN + BXA, ZMN + OTC) (Figure S7B, S8B). In general, combinational effects exceeded additive effects of the monotherapies (Figures S7A-B, S8A-B). Synergy evaluation was conducted using different reference models (Bliss, HSA, Loewe, ZIP) provided by the open-source web application SynergyFinder Plus [23]. We found strong synergistic effects for both drug combinations (Figures 5, S7, S8). The highest synergy scores of the individual drug combinations were calculated for 2.6 µM ZMN + 0.4 nM BXA and 0.7 µM ZMN + 0.5 µM OTC within all reference models, demonstrating the reliability of our findings (Figures S7G, S8G). The average synergistic effect of the different drug combinations was confirmed by the positive values of the overall synergy scores of all reference models (Figure S7H, S8H). These results indicate that ZMN has the potential to act synergistically with DAAs directed against IVs polymerase acidic protein (PA) and neuraminidase (NA).

## Discussion

Aquatic birds are the natural reservoir of AIVs. Due to spillover events from wild waterfowl into poultry, AIVs can acquire a multibasic cleavage site (MBCS) in the HA protein allowing for processing by ubiquitously expressed furin-like proteases and systemic spread [35]. Infections caused by these HPAIVs are associated with an increased pathogenicity in mammals [36,37]. Severe cases of HPAIV infection can induce multi-organ diseases affecting the lung, central nervous system or digestive system, resulting in acute respiratory distress syndrome (ARDS) and multi-organ failure, such as pulmonary haemorrhage, pneumothorax, pancytopenia, respiratory and renal failure, therefore presenting a substantial threat to public health [38–41]. While earlier clades had a high mortality rate of approximately 50% of human HPAIV infections, the latest reports show that the mortality rate of clade 2.3.4.4b is lower [4]. Since 2022, 67 cases of H5N1 clade 2.3.4.4b human infections have been reported for the U.S. [42], with additional 11 cases recorded for other countries [43]. While most cases were asymptomatic or mild, severe or critical illnesses, with one fatale case, were reported as well [43,44]. To treat human HPAIV infections, efficient treatment strategies are of great importance. Direct-acting antivirals (DAA) have a high potency in inhibiting viral replication. Nevertheless, in the past years, HPAIVs with reduced susceptibilities to DAAs like oseltamivir, zanamivir or baloxavir have occurred, increasing the risk that the drugs may be rendered ineffective [19,20,45,46]. An alternative treatment approach focuses on cellular factors important for viral replication, instead of targeting the virus itself [47]*.* In the past, we could show that the Raf/MEK/ERK signalling cascade is activated during human influenza A and B virus infections [48,49]. Inhibition of MEK1/2 with kinase-specific inhibitors efficiently reduces the release of progeny viral particles *in vitro* and *in vivo* [17,24,25,50]. In this study, we examined the inhibitory potency of the specific MEK1/2 inhibitor zapnometinib (ZMN) against HPAIVs with regard to the global spread of H5N1 clade 2.3.4.4b and the increasing number of spillover events reported in the last years.

To our knowledge, the current study first describes the promising anti-influenza properties of ZMN against HPAIVs in general, but especially against currently circulating H5Nx viruses. The significantly reduced viral titres of all H5Nx and H7Nx viruses caused by the ZMN treatment, which is comparable to the human PR8 (H1N1) strain, as well as the high SI values, confirm its potential to be used as an anti-HPAIV drug (Figure 1, 2; Table 1; Figures S1–S3). Importantly, these effects are independent of cell lines and virus subtypes and were also confirmed in primary cells (Figure 2, 4; Figure S2, 4A–C), excluding a cell line- or virus-specific effect.

The Raf/MEK/ERK pathway is utilized by influenza viruses in the late stage of the viral replication, to promote the nuclear export of newly synthesized vRNP complexes [48]. Recently we could show that not ERK itself but RSK1 has an important role within this process. It translocates into the nucleus to phosphorylate the two serine residues 269 and 392 in NP, allowing the binding of M1 to the vRNP complex, a crucial step in the formation of the vRNP export complexes [22]. In addition, the antiviral mode of action of MEK1/2 inhibitors has been unravelled for other viruses beyond IV. Preugschas *et al*., reported that the MEK1/2 inhibitor U0126 reduces the accumulation of the respiratory syncytial virus (RSV) F-protein at the plasma membrane of infected cells [51] while Albarnaz *et al*., described that MEK1/2 inhibition interferes with viral RNA replication of the yellow fever virus (YFV), leading to a reduced accumulation of the viral NS4A/B protein [52]. Within this study, we show that MEK1/2 inhibition results in nuclear retention of the vRNP complexes of all tested H5N1 and H7Nx viruses. In an alignment of sequences of human, avian, swine and bat IVs we found that almost all viruses do possess the serine residues 269 and 392 (Table S3), which were described as phosphorylation sites of RSK1, the downstream kinase of MEK/ERK [22]. This high level of conservation does not only highlight the important role of these sites for the virus life cycle of IV, but may also explain the overall anti-influenza properties of MEK1/2 and RSK1 inhibitors in general, as well as of ZMN in particular. Recent research has highlighted the possibility of pathway redundancies during viral infections, which would be detrimental to HTA therapies. To facilitate entry, SARS-CoV-2 appears to utilizes both, the host proteases TMPRSS2 or cathepsin [53] and HCV can adapt to use CLDN6 under selection pressure in CLDN1-knockout cells [54]. Yet, it is likely that viruses lack the flexibility to exploit alternative host factors compensating for the missing function during ZMN treatment, as we could not detect a reduced susceptibility to ZMN for SARS-CoV-2 after 30 passages *in vitro* [15]*,* a finding that is in line with previous data on IAV using other MEK1/2 inhibitors [49].

The synergistic effects observed in this study (Figure 5, S7, S8), offer a promising strategy for pandemic preparedness, by increasing the antiviral efficacy and reducing the likelihood of resistance development, while decreasing the concentrations of the single drugs and clinical side effects [55]. Given, that DAAs have to be administered very early after infection to be efficient, combinations with HTAs may have another benefit. Besides the direct antiviral effect, MEK1/2 inhibitors like ZMN, Trametinib or U0126 have the potential to attenuate virus-induced hyperinflammatory responses [16,25,56]. In the past, H5N1 infections in humans have rapidly progressed to these severe hyperinflammatory stages, where DAAs are not effective anymore. Thus, drugs or drug combinations with such a dual beneficial mode of action have the advantage that they can be administered during the acute viral infection phase, as well as the following hyperinflammatory phase. In that respect it is noteworthy that ZNM showed clinical efficacy in moderate to severely infected hospitalized patients (WHO Clinical severity status CSS = 4) [21], a patient cohort in which the DAA molnupiravir actually failed [57]. It is of course necessary to carefully balance between reducing harmful inflammation and maintaining critical immune functions, an evaluation that can only partially be addressed in *in vitro* and *in vivo* experiments. Thus, clinical trials are needed to further evaluate these findings. Immunomodulation might be problematic if therapy starts too early and combinational antiviral therapies might act antagonistic in patients. Furthermore, adverse side effects caused by complex drug–drug interactions need to be evaluated [58–60]. Despite these concerns, the combinational use of HTAs and DAAs could be an important countermeasure in managing future viral outbreaks.

A limitation of this study is that the findings were derived solely from *in vitro* experiments, with no additional *in vivo* or *ex vivo* data to support the observed effects. While *in vitro* studies provide valuable mechanistic insights, they do not fully mirror the complexity of a natural infection, where host immune responses and tissue-specific factors play crucial roles, or drug–drug interactions of combinational therapies might negatively influence the clinical outcome. Aspects such as bioavailability, metabolism and distribution *in vivo* can greatly affect the optimal therapeutic dose, complicating straightforward comparisons. ZMN, as a weak acid, exhibits reduced cell membrane permeability. Consequently, to gain sufficient intracellular drug levels to inhibit viral infections *in vitro,* higher concentrations are necessary compared to its parental drug CI-1040. In contrast, lower *in vivo* concentrations are needed due to its better bioavailability [17]. This is also reflected by the results of two phase 1 clinical trials in 2019 (NCT04385420) and 2023 (NCT05555823), demonstrating that the drug is safe and very well tolerated in humans. Thus, the drug can safely be administered in effective concentrations reflecting the clinical relevance.

In summary, our results give important insights into the susceptibility of the H5Nx clade 2.3.4.4b viruses to MEK1/2 inhibition via ZMN.

While it was already shown, that ZMN acts antiviral against human IVs, here we show for the first time that it displays antiviral properties against HPAIVs of the clade 2.3.4.4b. These results might open new options in the treatment of human infections with HPAIVs in a stand-alone as well as combinational treatment with DAAs, especially in a scenario of increasing levels of resistance against current DAAs in the future.

## Supplementary Material

Supplemental Material

## Data Availability

The authors confirm that the data supporting the findings of this study are available within the article and its supplementary materials.

## References

[1] Blagodatski A, Trutneva K, Glazova O, et al. Avian influenza in wild birds and poultry: dissemination pathways, monitoring methods, and virus ecology. Pathogens. 2021;10:1–23. doi:10.3390/pathogens10050630 PMC8161317 34065291

[2] Franca MS, Brown JD. Influenza pathobiology and pathogenesis in avian species. In: Compans R, Oldstone M, editors. Influ pathog control - Vol I curr top microbiol immunol. Cham: Springer; 2014. p. 221–242. doi:10.1007/82_2014_385 25015786

[3] Duan L, Bahl J, Smith GJD, et al. The development and genetic diversity of H5N1 influenza virus in China, 1996-2006. Virology. 2008;380:243–254. doi:10.1016/j.virol.2008.07.038 18774155 PMC2651962

[4] WHO. Avian influenza weekly update number 983. Human infection with avian influenza A(H5) viruses. Regional Office for the Western Pacific. 2024;983:1–4.

[5] Caliendo V, Lewis NS, Pohlmann A, et al. Transatlantic spread of highly pathogenic avian influenza H5N1 by wild birds from Europe to North America in 2021. Sci Rep. 2022;12:1–18. doi:10.1038/s41598-022-13447-z 35821511 PMC9276711

[6] Kandeil A, Patton C, Jones JC, et al. Rapid evolution of A(H5N1) influenza viruses after intercontinental spread to North America. Nat Commun. 2023;14:1–13. 37248261 10.1038/s41467-023-38415-7 PMC10227026

[7] Agüero M, Monne I, Azucena Sánchez O, et al. Highly pathogenic avian influenza A(H5N1) virus infection in farmed minks, Spain, October 2022. Eurosurveillance. 2023;3:28. doi:10.2807/1560-7917.ES.2023.28.3.2300001 PMC9853945 36695488

[8] Leguia M, Garcia-Glaessner A, Muñoz-Saavedra B, et al. Highly pathogenic avian influenza A (H5N1) in marine mammals and seabirds in Peru. Nat Commun. 2023;14:1–11. doi:10.1038/s41467-023-41182-0 37679333 PMC10484921

[9] Burrough ER, Magstadt DR, Petersen B, et al. Highly pathogenic avian influenza A(H5N1) clade 2.3.4.4b virus infection in domestic dairy cattle and cats, United States, 2024. Emerg Infect Dis. 2024;30:1335–1343. doi:10.3201/eid3007.240508 38683888 PMC11210653

[10] Mirolo M, Pohlmann A, Ahrens AK, et al. Highly pathogenic avian influenza A virus (HPAIV) H5N1 infection in two European grey seals (halichoerus grypus) with encephalitis. Emerg Microbes Infect. 2023;12:1–9. doi:10.1080/22221751.2023.2257810 PMC10768861 37682060

[11] Perwitasari O, Yan X, Johnson S, et al. Targeting organic anion transporter 3 with probenecid as a novel anti-influenza A virus strategy. Antimicrob Agents Chemother. 2013;57:475–483. doi:10.1128/AAC.01532-12 23129053 PMC3535968

[12] Triana-Baltzer GB, Babizki M, Chan MCW, et al. DAS181, a sialidase fusion protein, protects human airway epithelium against influenza virus infection: an in vitro pharmacodynamic analysis. J Antimicrob Chemother. 2009;65:275–284. doi:10.1093/jac/dkp421 19942616 PMC2809246

[13] Moss RB, Hansen C, Sanders RL, et al. A phase II study of DAS181, a novel host directed antiviral for the treatment of influenza infection. J Infect Dis. 2012;206:1844–1851. doi:10.1093/infdis/jis622 23045618 PMC3570175

[14] Pizzorno A, Terrier O, de Lamballerie CN, et al. Repurposing of drugs as novel influenza inhibitors from clinical gene expression infection signatures. Front Immunol. 2019;10:1–17. doi:10.3389/fimmu.2019.00060 30761132 PMC6361841

[15] Schreiber A, Rodner F, Oberberg N, et al. The host-targeted antiviral drug zapnometinib exhibits a high barrier to the development of SARS-CoV-2 resistance. Antiviral Res. 2024;225:1–10. doi:10.1016/j.antiviral.2024.105840 38438015

[16] Schreiber A, Viemann D, Schöning J, et al. The MEK1/2-inhibitor ATR-002 efficiently blocks SARS-CoV-2 propagation and alleviates pro-inflammatory cytokine/chemokine responses. Cell Mol Life Sci. 2022;79:1–18. doi:10.1007/s00018-021-04085-1 PMC8747446 35013790

[17] Laure M, Hamza H, Koch-Heier J, et al. Antiviral efficacy against influenza virus and pharmacokinetic analysis of a novel MEK-inhibitor, ATR-002, in cell culture and in the mouse model. Antiviral Res. 2020;178:104806. doi:10.1016/j.antiviral.2020.104806 32304723

[18] Schreiber A, Ambrosy B, Planz O, et al. The MEK1/2 inhibitor ATR-002 (zapnometinib) synergistically potentiates the antiviral effect of direct-acting anti-SARS-CoV-2 drugs. Pharmaceutics. 2022;14:1–12. doi:10.3390/pharmaceutics14091776 PMC9506552 36145524

[19] Kode SS, Pawar SD, Tare DS, et al. A novel I117 T substitution in neuraminidase of highly pathogenic avian influenza H5N1 virus conferring reduced susceptibility to oseltamivir and zanamivir. Vet Microbiol. 2019;235:21–24. doi:10.1016/j.vetmic.2019.06.005 31282375

[20] Nguyen HT, Chesnokov A, De La Cruz J, et al. Antiviral susceptibility of clade 2.3.4.4b highly pathogenic avian influenza A(H5N1) viruses isolated from birds and mammals in the United States, 2022. Antiviral Res. 2023;217:105679. doi:10.1016/j.antiviral.2023.105679 37494978 PMC10508830

[21] Rohde G, Stenglein S, Prozesky H, et al. Efficacy and safety of zapnometinib in hospitalised adult patients with COVID-19 (RESPIRE): a randomised, double-blind, placebo-controlled, multicentre, proof-of-concept, phase 2 trial. eClinicalMedicine. 2023;65:102237. doi:10.1016/j.eclinm.2023.102237 38106555 PMC10725048

[22] Schreiber A, Boff L, Anhlan D, et al. Dissecting the mechanism of signaling-triggered nuclear export of newly synthesized influenza virus ribonucleoprotein complexes. Proc Natl Acad Sci U S A. 2020;117:16557–16566. doi:10.1073/pnas.2002828117 32601201 PMC7368312

[23] Zheng S, Wang W, Aldahdooh J, et al. Synergyfinder plus: toward better interpretation and annotation of drug combination screening datasets. genomics. Proteomics Bioinforma. 2022;20:587–596. doi:10.1016/j.gpb.2022.01.004 PMC9801064 35085776

[24] Haasbach E, Müller C, Ehrhardt C, et al. The MEK-inhibitor CI-1040 displays a broad anti-influenza virus activity in vitro and provides a prolonged treatment window compared to standard of care in vivo. Antiviral Res. 2017;142:178–184. doi:10.1016/j.antiviral.2017.03.024 28377100

[25] Schräder T, Dudek SE, Schreiber A, et al. The clinically approved MEK inhibitor trametinib efficiently blocks influenza A virus propagation and cytokine expression. Antiviral Res. 2018;157:80–92. doi:10.1016/j.antiviral.2018.07.006 29990517

[26] Haasbach E, Hartmayer C, Planz O. Combination of MEK inhibitors and oseltamivir leads to synergistic antiviral effects after influenza A virus infection in vitro. Antiviral Res. 2013;98:319–324. doi:10.1016/j.antiviral.2013.03.006 23523553

[27] Park J-G, Ávila-Pérez G, Nogales A, et al. Identification and characterization of novel compounds with broad-spectrum antiviral activity against influenza A and B viruses. J Virol. 2020;94:1–20. doi:10.1128/jvi.02149-19 PMC7081893 31941776

[28] Xiong R, Zhang L, Li S, et al. Novel and potent inhibitors targeting DHODH are broad-spectrum antivirals against RNA viruses including newly-emerged coronavirus SARS-CoV-2. Protein Cell. 2020;11:723–739. doi:10.1007/s13238-020-00768-w 32754890 PMC7402641

[29] Kumar N, Sharma NR, Ly H, et al. Receptor tyrosine kinase inhibitors that block replication of influenza A and other viruses. Antimicrob Agents Chemother. 2011;55:5553–5559. doi:10.1128/AAC.00725-11 21930873 PMC3232778

[30] Hu J, Ma L, Wang H, et al. A novel benzo-heterocyclic amine derivative N30 inhibits influenza virus replication by depression of inosine-5’-monophospate dehydrogenase activity. Virol J. 2017;14:1–9. doi:10.1186/s12985-017-0724-6 28298229 PMC5353780

[31] Denisova O V, Sod̈erholm S, Virtanen S, et al. Akt inhibitor MK2206 prevents influenza pH1N1 virus infection in vitro. Antimicrob Agents Chemother. 2014;(58):3689–3696. doi:10.1128/AAC.02798-13 24752266 PMC4068572

[32] Perwitasari O, Johnson S, Yan X, et al. Verdinexor, a novel selective inhibitor of nuclear export, reduces influenza A virus replication in vitro and in vivo . J Virol. 2014;88:10228–10243. doi:10.1128/jvi.01774-14 24965445 PMC4136318

[33] Taniguchi K, Ando Y, Kobayashi M, et al. Characterization of the In vitro and In vivo efficacy of baloxavir marboxil against H5 highly pathogenic avian influenza virus infection. Viruses. 2022;14:1–19. doi:10.3390/v14010111 PMC8777714 35062315

[34] Leneva IA, Roberts N, Govorkova EA, et al. The neuraminidase inhibitor GS4104 (oseltamivir phosphate) is efficacious against A/Hong Kong/156/97 (H5N1) and A/Hong Kong/1074/99 (H9N2) influenza viruses. Antiviral Res. 2000;48:101–115. doi:10.1016/S0166-3542(00)00123-6 11114412

[35] Horimoto T, Kawaoka Y. Reverse genetics provides direct evidence for a correlation of hemagglutinin cleavability and virulence of an avian influenza A virus. J Virol. 1994;68:3120–3128. doi:10.1128/jvi.68.5.3120-3128.1994 8151777 PMC236802

[36] Schrauwen EJA, Herfst S, Leijten LM, et al. The multibasic cleavage site in H5N1 virus Is critical for systemic spread along the olfactory and hematogenous routes in ferrets. J Virol. 2012;86:3975–3984. doi:10.1128/jvi.06828-11 22278228 PMC3302532

[37] Suguitan AL, Matsuoka Y, Lau Y-F, et al. The multibasic cleavage site of the hemagglutinin of highly pathogenic A/Vietnam/1203/2004 (H5N1) avian influenza virus acts as a virulence factor in a host-specific manner in mammals. J Virol. 2012;86:2706–2714. doi:10.1128/jvi.05546-11 22205751 PMC3302284

[38] Imperia E, Bazzani L, Scarpa F, et al. Avian influenza: could the H5N1 virus Be a potential next threat? Microbiol Res (Pavia. 2023;14:635–645. doi:10.3390/microbiolres14020045

[39] Mehta K, Goneau LW, Wong J, et al. Zoonotic influenza and human health—part 2: clinical features, diagnosis, treatment, and prevention strategies. Curr Infect Dis Rep. 2018;20(10):38. doi:10.1007/s11908-018-0643-8 30069787 PMC7102074

[40] Lai S, Qin Y, Cowling BJ, et al. Global epidemiology of avian influenza A H5N1 virus infection in humans, 1997-2015: A systematic review of individual case data. Lancet Infect Dis. 2016;16:e108–e118. doi:10.1016/S1473-3099(16)00153-5 27211899 PMC4933299

[41] Kalthoff D, Globig A, Beer M. (Highly pathogenic) avian influenza as a zoonotic agent. Vet Microbiol. 2010;140:237–245. doi:10.1016/j.vetmic.2009.08.022 19782482

[42] CDC. CDC - First H5 Bird Flu Death Reported in United States [Internet]. 2025 [cited 2025 Jan 13]. Available from: https://www.cdc.gov/media/releases/2025/m0106-h5-birdflu-death.html.

[43] Garg S, Reinhart K, Couture A, et al. Highly pathogenic avian influenza A(H5N1) virus infections in humans. N Engl J Med. 2024;392:1–12. doi:10.1056/NEJMoa2414610 39740051

[44] CDC. Technical Report: June 2024 Highly Pathogenic Avian Influenza A(H5N1) Viruses [Internet]. [cited 2024 Sep 12]. Available from: https://www.cdc.gov/bird-flu/php/technical-report/h5n1-06052024.html#cdc_research_or_data_summary_resources-resources.

[45] Earhart KC, Elsayed NM, Saad MD, et al. Oseltamivir resistance mutation N294S in human influenza A(H5N1) virus in Egypt. J Infect Public Health. 2009;2:74–80. doi:10.1016/j.jiph.2009.04.004 20701864

[46] Le QM, Kiso M, Someya K, et al. Isolation of drug-resistant H5N1 virus. Nature. 2005;437:1108. doi:10.1038/4371108a 16228009

[47] Lee SMY, Yen HL. Targeting the host or the virus: current and novel concepts for antiviral approaches against influenza virus infection. Antiviral Res. 2012;96:391–404. doi:10.1016/j.antiviral.2012.09.013 23022351 PMC7132421

[48] Pleschka S, Wolff T, Ehrhardt C, et al. Influenza virus propagation is impaired by inhibition of the Raf/MEK/ERK signalling cascade. Nat Cell Biol. 2001;3:301–305. doi:10.1038/35060098 11231581

[49] Ludwig S, Wolff T, Ehrhardt C, et al. MEK inhibition impairs influenza B virus propagation without emergence of resistant variants. FEBS Lett. 2004;561:37–43. doi:10.1016/S0014-5793(04)00108-5 15013748

[50] Droebner K, Pleschka S, Ludwig S, et al. Antiviral activity of the MEK-inhibitor U0126 against pandemic H1N1v and highly pathogenic avian influenza virus in vitro and in vivo. Antiviral Res. 2011;92:195–203. doi:10.1016/j.antiviral.2011.08.002 21854809

[51] Preugschas HF, Hrincius ER, Mewis C, et al. Late activation of the Raf/MEK/ERK pathway is required for translocation of the respiratory syncytial virus F protein to the plasma membrane and efficient viral replication. Cell Microbiol. 2019;21:1–14. doi:10.1111/cmi.12955 30223301

[52] Albarnaz JD, De Oliveira LC, Torres AA, et al. MEK/ERK activation plays a decisive role in yellow fever virus replication: implication as an antiviral therapeutic target. Antiviral Res. 2014;111:82–92. doi:10.1016/j.antiviral.2014.09.004 25241249

[53] Vanslambrouck JM, Neil JA, Rudraraju R, et al. Kidney organoids reveal redundancy in viral entry pathways during ACE2-dependent SARS-CoV-2 infection. J Virol. 2024;98:1–16. doi:10.1128/jvi.01802-23 PMC10949421 38334329

[54] Hopcraft SE, Evans MJ. Selection of a hepatitis C virus With altered entry factor requirements reveals a genetic interaction between the E1 glycoprotein and claudins. Physiol Behav. 2015;62:1059–1069. doi:10.1002/hep.27815 PMC4587996 25820616

[55] Wagoner J, Herring S, Hsiang T-Y, et al. Combinations of host- and virus-targeting antiviral drugs confer synergistic suppression of SARS-CoV-2. Microbiol Spectr. 2022;10:1–15. doi:10.1128/spectrum.03331-22 PMC9718484 36190406

[56] Pinto R, Herold S, Cakarova L, et al. Inhibition of influenza virus-induced NF-kappaB and Raf/MEK/ERK activation can reduce both virus titers and cytokine expression simultaneously in vitro and in vivo. Antiviral Res. 2011;92:45–56. doi:10.1016/j.antiviral.2011.05.009 21641936

[57] Arribas JR, Bhagani S, Lobo SM, et al. Randomized trial of molnupiravir or placebo in patients hospitalized with COVID-19. NEJM Evid. 2022;1:1–13. doi:10.1056/evidoa2100044 38319178

[58] Saravolatz LD, Winslow DL, Collins G, et al. Zidovudine alone or in combination with didanosine or zalcitabine in HIV-infected patients with the acquired immunodeficiency syndrome or fewer than 200 CD4 cells per cubic millimeter. N Engl J Med. 1996;335:1099–1106. doi:10.1056/NEJM19961010335150 8813040

[59] Hochster H, Dieterich D, Bozzette S, et al. Toxicity of combined ganciclovir and zidovudine for cytomegalovirus disease associated with AIDS: An AIDS clinical trials group study. Ann Intern Med. 1990;113:111–117. doi:10.7326/0003-4819-113-2-111 2163228

[60] Taburet AM, Singlas E. Drug interactions with antiviral drugs. Clin Pharmacokinet. 1996;30:385–401. doi:10.2165/00003088-199630050-00005 8743337

