# Supplementary material for: Influenza H5Nx viruses are susceptible to MEK1/2 inhibition by zapnometinib
Source: Emerg Microbes Infect. 2025 Feb 20;14(1):2471022. doi: 10.1080/22221751.2025.2471022 (PMC11915740; doi:10.1080/22221751.2025.2471022)
Supplement: Supplemental Material [file TEMI_A_2471022_SM6140.docx]

Taylor & Francis Word Template for journal articles

**Supplementary data**

André Schreiber^a^, Nicole Oberberg^a^, Benjamin Ambrosy^a^, Franziska Rodner^a^, Sriram Kumar^a^, Duygu Merve Caliskan^a^, Linda Brunotte^a^, Martin Beer^b^, Stephan Ludwig^a^*

^a^Institute of Virology Muenster, University of Muenster, Muenster, Germany

^b^Institute of Diagnostic Virology, Friedrich-Loeffler-Institut, Greifswald-Insel Riems, Germany.

SK, DMC and SL are members of the EvoPAD Research Training Group 2220, University of Muenster, Muenster, Germany

*Institute of Virology, Von-Esmarch Str. 56, 48149, Muenster, Germany.

ludwigs@uni-muenster.de

**Influenza H5Nx Viruses are susceptible to MEK1/2 Inhibition via zapnometinib**

Figure S1. Reduced production of H5N1 progeny virus particles after ZMN treatment.

*(Related to Figure 1)*

(A, B) A549 cells were infected with different H5N1 variants or strain PR8 (H1N1) (MOI: 0.01). At 0.5 h.p.i. ZMN treatment was initiated. Untreated (Virus) and DMSO treated cells served as negative controls. At 24 h.p.i. viral titers were analyzed via plaque titration. (A) Viral titers in PFU/ml. (B) Viral titers in % of DMSO. DMSO was arbitrarily set to 100 %.

(C, D) A549 cells were treated with increasing concentrations of ZMN (0.39 – 400 µM). At 24 h and 48 h post treatment cell viability was determined using the LDH assay. *See also Table S1.*

(A-D) Data represent means ± SEM of three independent experiments, each performed in triplicates. Significance was calculated using a one-way ANOVA in combination with a Dunnett´s multiple comparisons test with DMSO as reference (** p ≤ 0.0021; *** p ≤ 0.0002; **** p ≤ 0.0001).

Figure S2. Inhibitory effect of ZMN on H5Nx viruses. *(Related to Figure 2)*

H5Nx viruses were used to infect A549 cells (MOI: 0.01). At 0.5 h.p.i. ZMN treatment was initiated.

Viral titers were analyzed 24 h.p.i. Shown are PFU/ml. Data represent means ± SEM of three independent experiments, each performed in triplicates. Significance was calculated using a one-way ANOVA in combination with a Dunnett´s multiple comparisons test with DMSO as reference for each virus individually (** p ≤ 0.0021; *** p ≤ 0.0002; **** p ≤ 0.0001).

Figure S3. Inhibitory effect of ZMN on H7Nx viruses. *(Related to Figure 3)*

(A) A549 cells were infected with different H7Nx viruses (MOI: 5). At 3 h.p.i. cells were treated with ZMN (50 µM). Samples were prepared for immunofluorescence analysis 9 h.p.i. NP protein and PB2-vRNA are stained. Shown are representative images of three independent experiments. (Scale bar: 20 µm)

(B, C) A549 cells were infected with different H7Nx viruses (MOI: 0.01). At 0.5 h.p.i. cells were treated with ZMN. At 24 h.p.i. viral titers were analyzed via plaque titration. Shown are results of three independent experiments, each performed in triplicates. Significance was calculated using a one-way ANOVA in combination with a Dunnett´s multiple comparisons test with DMSO as reference for each virus individually (* p ≤ 0.0332; ** p ≤ 0.0021; *** p ≤ 0.0002; **** p ≤ 0.0001). (B) Shown are PFU/ml. (C) Shown are % of DMSO. DMSO was arbitrarily set to 100 %.

Figure S4. ZMN acts antiviral against Thailand (H5N1) and Chicken (H5N1) in different cell lines. *(Related to Figure 4)*

(A-C) Different cell lines (A, B) or HBEpCs (C) were infected with Thailand (H5N1) (A, B: MOI: 0.01; C: MOI: 0.1) or Chicken (H5N1) (C: MOI: 0.1). At 0.5 h.p.i. cells were treated with ZMN. At 24 h.p.i. viral titers were analyzed via plaque titration. (A, C) Absolute viral titers in PFU/ml. (B) Relative viral titers in % of DMSO. DMSO was arbitrarily set to 100 %. *See also Table S2.*

(D, E) Cell lines (D) or HBEpCs (E) were treated with ZMN (D: 0.39 – 400 µM; E: 50 µM). At 24 h post treatment cell viability was determined using the LDH assay. *See also Table S1.*

(A-E) Shown are results of three independent experiments, each performed in triplicates. (A-C) Significance was calculated using a one-way ANOVA in combination with a Dunnett´s multiple comparisons test with DMSO as reference (* p ≤ 0.0332; ** p ≤ 0.0021; *** p ≤ 0.0002; **** p ≤ 0.0001).

Figure S5. Structural positions of the amino acid residues S269/S392 and S327/S448 in influenza A and B nucleoproteins.

Depicted are the localizations of the amino acid residues S269/S392 (IAV) and S327/S448 (IBV) within NP of the influenza A viruses A/HK/483/97 (H5N1) (pdb: 2q06), A/WSN/1933 (H1N1) (pdb: 4dyb), A/Puerto Rico/8/1934 (PR8) (H1N1) (pdb: 2ymn) and the influenza B virus B/Managua/4577.01/2008 (pdb: 3tj0).

Figure S6. Antiviral activity of BXA and OTC against Chicken (H5N1). *(Related to Figure 5)*

(A-F) A549 cells were infected with Chicken (H5N1) (MOI: 0.01). 0.5 h.p.i. drug treatment was initiated.

Viral titers were analyzed 24 h.p.i. and are depicted as PFU/ml (A, D) or % of DMSO control, with DMSO arbitrarily set to 100 %. (B, E). Calculation of EC values (C, F).

(G, H) A549 cells were treated with increasing concentrations of BXA (0.01 – 3000 nM) or OTC (0.05 – 2000 µM).

(I, J) A549 cells were treated with the drug combinations ZMN + BXA or ZMN + OTC using increasing concentrations. DMSO (BXA) or ddH_2_O/DMSO (OTC) served as negative controls.

(G-J) At 24 h post treatment cell viability was determined using the LDH assay.

Data represent means ± SEM of three independent experiments, each performed in triplicates. Significance was calculated using a one-way ANOVA in combination with a Dunnett´s multiple comparisons test with DMSO (BXA, ZMN + BXA), ddH2O (OTC) or ddH2O/DMSO (ZMN + OTC) as references (* p ≤ 0.0332; ** p ≤ 0.0021; *** p ≤ 0.0002; **** p ≤ 0.0001).

Figure S7: Determination of the ZMN + BXA synergism. *(Related to Figure 5)*

Chicken (H5N1) (MOI: 0.01) was used to infect A549 cells. 0.5 h.p.i. drug treatment was initiated. Untreated (Chicken) and DMSO-treated (0 nM BXA, 0 µM ZMN) cells served as controls. 24 h.p.i. viral titers were determined by plaque titration.

(A, B) Viral titers of the combinational drug treatments in PFU/ml (A) or % of DMSO (B). Data represent means ± SEM of three independent experiments, each performed in triplicates. (B) 10 %, 50 % or 90 % titer reductions are indicated by the dashed lines.

(C) Dose-response curves of single treatments.

(D) 2D dose response matrix of the drug combination ZMN + BXA.

(E) Landscape visualization of the 2D contour visualization shown in Figure 5A.

(F) 2D heat maps of the 2D contour visualization shown in Figure 5A.

(E, F) Color coded visualization with red indicating synergistic interactions while green indicates antagonistic interactions.

(G) Synergy values of the ZMN + BXA drug combinations calculated with the indicated synergy reference models.

(H) Synergy scores of the ZMN + BXA drug combinations evaluated with the shown synergy reference models.

Figure S8: Determination of the ZMN + OTC synergism. *(Related to Figure 5)*

Chicken (H5N1) (MOI: 0.01) was used to infect A549 cells. 0.5 h.p.i. drug treatment was initiated. Untreated (Chicken) and DMSO-treated (0 µM OTC, 0 µM ZMN) cells served as controls. 24 h.p.i. viral titers were determined by plaque titration.

(A, B) Viral titers of the combinational drug treatments in PFU/ml (A) or % of DMSO (B). Data represent means ± SEM of three independent experiments, each performed in triplicates. (B) 10 %, 50 % or 90 % titer reductions are indicated by the dashed lines.

(C) Dose-response curves of single treatments.

(D) 2D dose response matrix of the drug combination ZMN + OTC.

(E) Landscape visualization of the 2D contour visualization shown in Figure 5B.

(F) 2D heat maps of the 2D contour visualization shown in Figure 5B.

(G) Synergy values of the ZMN + OTC drug combinations calculated with the indicated synergy reference models.

(H) Synergy scores of the ZMN + OTC drug combinations evaluated with the shown synergy reference models.

Table S1. ZMN concentrations tested for cytotoxicity. *(Related to Figures S1C, D and S4D)*

The indicated concentrations are shown in µM.

| **1.** | **2.** | **3.** | **4.** | **5.** | **6.** | **7.** | **8.** | **9.** | **10.** | **11.** |
| --- | --- | --- | --- | --- | --- | --- | --- | --- | --- | --- |
| 0.39 | 0.78 | 1.56 | 3.12 | 6.25 | 12.5 | 25 | 50 | 100 | 200 | 400 |

Table S2. Relative viral titers after ZMN treatment in different cell lines. *(Related to Figure 4B and Figure S4B)*

Shown are viral titers in percentage related to the DMSO controls after ZMN treatment.

| **Virus** | **Conc._ZMN_ [µM]** | **A549** | **Calu-3** | **CaCo2** | **MDCK II** | **VeroE6** |
| --- | --- | --- | --- | --- | --- | --- |
| Chicken (H5N1) | 0.78 | 88.70  ± 9.64 | 78.28  ± 5.17 | 81.50  ± 3.37 | 89.25  ± 3.91 | 94.85  ± 5.46 |
|  | 3.12 | 43.93  ± 6.87 | 54.21  ± 4.08 | 36.99  ± 1.11 | 53.81  ± 2.34 | 71.57  ± 15.11 |
|  | 25 | 9.15  ± 1.18 | 9.75  ± 6.25 | 14.55  ± 2.73 | 7.18  ± 6.21 | 19.87  ± 14.26 |
| Thailand (H5N1) | 0.78 | 94.82  ± 0.96 | 86.95  ± 8.02 | 85.68  ± 1.71 | 87.00  ± 14.09 | 99.13  ± 16.37 |
|  | 3.12 | 52.87  ± 1.32 | 56.49  ± 1.48 | 41.90  ± 7.00 | 43.44  ± 3.19 | 62.27  ± 12.79 |
|  | 25 | 7.49  ± 1.82 | 7.66  ± 1.31 | 9.92  ± 0.81 | 7.06  ± 1.79 | 22.06  ± 2.61 |

Table S3. Polymorphisms of the NP residues S269/S392 in influenza A viruses and S327/S448 in influenza B viruses.

NP sequences of human, avian, swine and bat influenza A viruses and influenza B viruses were analyzed. Number of aligned sequences per virus, polymorphisms and virus subtype are indicated. Alignment was performed using the NCBI Influenza Database Nucleotide BLASTp alignment tool.

| **Virus** | **Sequences** | **Polymorphisms S269 / S392** | **Subtype** |
| --- | --- | --- | --- |
| **Human** |  |  |  |
| H1N1 | 17337 | S269T | A/Mexico/UASLP-006/2008 |
| H3N2 | 31301 | - |  |
| **Avian** |  |  |  |
| H1N1 | 605 | - |  |
| H5N1 | 2637 | - |  |
| H5N2 | 1351 | S269A | A/Chicken/Durango/1433-8/2005 |
| H5N3 | 154 | - |  |
| H5N4 | 6 | - |  |
| H5N5 | 50 | - |  |
| H5N6 | 675 | - |  |
| H5N7 | 24 | - |  |
| H5N8 | 590 | - |  |
| H5N9 | 36 | S269A | A/Glaucous gull/Wisconsin/486108-4/2007 |
| H7N7 | 278 | - |  |
| H7N9 | 804 | S269T | A/Blue-winged teal/Guatemala/CIP049-02/2008 |
| H9N2 | 3843 | S269A | A/Chicken/Bangladesh/11154/2011 |
| **Swine** |  |  |  |
| H1N1 | 4008 | - |  |
| H3N2 | 3334 | S269A | A/Swine/Mexico/AVX32/2012 |
|  |  |  |  |
| **Bat** |  |  |  |
| H18N11 | 4 | S269A | A/Artibeus lituratus/Brazil/2301/2012  A/Artibeus lituratus/Brazil/2344/2012  A/Dark fruit-eating bat/Bolivia/PBV780-781/2011  A/Flat-faced bat/Peru/033/2010 |
|  |  |  |  |
| **Influenza B** | **Sequences** | **Polymorphisms S327 / S448** | **Virus** |
|  | 11976 | *S448X* | B/Mississippi/UR06-0408/2007 |
